# Hydrogen motion in rutile TiO_2_

**DOI:** 10.1038/s41598-017-16660-3

**Published:** 2017-12-06

**Authors:** A. J. Hupfer, E. V. Monakhov, B. G. Svensson, I. Chaplygin, E. V. Lavrov

**Affiliations:** 1 University of Oslo, Physics Department/Center for Materials Science and Nanotechnology, P.O. Box 1048 Blindern, Oslo, N-0316 Norway; 2 Technische Universität Dresden, 01062 Dresden, Germany

## Abstract

Uniaxial-stress experiments have been performed for the 3287- and 2445-cm^−1^ local vibrational modes assigned to the positive charge state of interstitial hydrogen $({{\rm{H}}}_{i}^{+})$ and deuterium $({{\rm{D}}}_{i}^{+})$, respectively, occurring in mono-crystalline rutile TiO2. The onset of the defect alignment under the stress applied perpendicular to the [001] axis is detected at 165 K (185 K), which corresponds to the activation energy of 0.53 eV (0.58 eV) for interstitial hydrogen (deuterium). Based on these findings the diffusion constants of ${{\rm{H}}}_{i}^{+}$ and ${{\rm{D}}}_{i}^{+}$ along the [001] axis of TiO2 are determined. The experimental data are complemented by density-functional theory calculations and compared with the earlier results on the diffusion of ${{\rm{H}}}_{i}^{+}$/${{\rm{D}}}_{i}^{+}$ at elevated temperatures up to 700 °C. It is found that the activation energy value deduced from our low-temperature stress measurements yields a very good agreement with the high-temperature data, covering a dynamic range of 12 orders of magnitude.

## Introduction

Hydrogen is an ubiquitous impurity in many solids and particularly in oxides. It strongly affects the electronic and structural properties of these materials. Interstitial hydrogen can act either as an amphoteric impurity, giving rise to deep levels in the band gap with positive, neutral, and negative charge states, or it can form a level at the conduction band edge and act as a donor^1–5^. The latter is the case in titanium dioxide or titania (TiO_2_).

An enhancement of the *n*-type conductivity of rutile polymorph of TiO_2_ after electrolytically induced hydrogenation was reported already in 1963^6^. There, interstitial hydrogen (H_*i*_) forms a single dative bond to oxygen and is located in the open ***c*** channel of the crystal^7,8^. While there is a general consensus about the microscopic structure of H_*i*_ in rutile TiO_2_, its electrical activity is still not quite well understood. Earlier theoretical models predicted a shallow donor behavior^9,10^, whereas more recent first principles calculations assign it to a deep donor because of the strong electron-lattice interaction and formation of small polaron localized at the nearby Ti atom^11,12^.

Interstitial hydrogen in rutile TiO_2_ results in a stretch local vibrational mode (LVM) at around 3290 cm^−1^ ^13,14^ which was recently shown to consist of at least three modes assigned to the positive (one) and neutral (two or three) charge states of H_*i*_^15^. Whereas the number of LVMs due to the neutral species remains controversial^16^, there is a general agreement that vibrational modes of ${{\rm{H}}}_{i}^{+}$ and ${{\rm{D}}}_{i}^{+}$ at helium temperatures have frequencies 3287 and 2445 cm^−1^, respectively^15,16^.

The diffusion of hydrogen in rutile TiO_2_ is highly anisotropic with the activation barrier being much lower in the ***c***-direction^17^. Interestingly, a giant enhancement of the diffusivity was observed upon electron irradiation^18^ and infrared illumination^19^. The data on the diffusivity of hydrogen isotopes in bulk rutile TiO_2_ are controversial. Johnson *et al*. investigated the diffusion of hydrogen and deuterium at temperatures from 350 to 700 °C and found that the diffusion of both isotopes obeys the Fick’s law with an activation energy of 0.58 eV in the *c*-direction, the ratio of pre-exponential factors being consistent with the classical inverse square root dependence on isotope mass^17^. The diffusion of tritium was investigated by Caskey^20^ at temperatures between 155 and 300 and by Cathcart *et al*.^21^ at temperatures between 250 and 700 °C. Whereas the value of 0.39 eV for the activation energy of the diffusion in ***c***-direction was given by the former author, the latter reported an almost two times higher value of 0.75 eV.

Here we address the issue of hydrogen diffusion in rutile TiO_2_ by studying the stress-induced dichroism of the LVMs due to ${{\rm{H}}}_{i}^{+}$ and ${{\rm{D}}}_{i}^{+}$. Regardless of the actual ionization energy of the electron polaron bound to H_*i*_, these donors are ionized at elevated temperatures which implies that primarily the positive charge state should be looked at in order to investigate the diffusion process of interstitial hydrogen in TiO_2_.

The advantage of stress-induced dichroism stems from the fact that it allows to probe an elementary diffusion step—a jump between the two neighboring lattice sites. Indeed similar experiments performed for hydrogen-related defects in Si^22_24^, GaAs^25,26^, ZnO^27,28^, and very recently In_2_O_3_^29^ provided a great deal of insight in the kinetics of hydrogen motion in these semiconductors.

Here, we will show that stress applied along the [100] crystallographic direction of rutile lifts the orientational degeneracy between the four equivalent positions of hydrogen in the *c* channel of the lattice. Subsequent annealing under the stress at elevated temperature results in the alignment of the O–H bonds in the crystal in accordance with the Boltzmann distribution. The observed thermalization process enables determination of the activation energy for the diffusion process along the ***c*** channel of rutile TiO_2_.

## Methods

### Experimental details

The rutile TiO_2_ samples employed in this study were float-zone grown wafers purchased from MTI Corp. Hydrogen and deuterium were introduced by annealing in a H_2_O and D_2_O mixture in oxygen ambient used as a carrier gas. The annealings were carried out at a temperature of 1100 °C within four hours. The sample that revealed the strongest ${{\rm{H}}}_{i}^{+}$ and ${{\rm{D}}}_{i}^{+}$ signals with only trace intensities due to the neutral charge state of the species was chosen for the uniaxial stress measurements. It was cut to the size 1 × 2 × 5 mm^3^ parallel to the [001] (*c*), [010] (*b*), and [100] (*a*) crystallographic directions, respectively.

Uniaxial stress measurements were performed in a home-built stress rig mounted in a gas-flow cryostat equipped with ZnSe windows. The stress was supplied by a pneumatic cylinder and transferred via a push rod to the sample.

The infrared absorption spectra were recorded with a Bomem DA3.01 Fourier transform spectrometer equipped with a globar light source, a KBr beamsplitter and a liquid-nitrogen-cooled InSb detector. The spectra were measured at *T* ≤ 10 K, unless mentioned otherwise, with the ***c*** axis of the sample aligned parallel to the incoming beam, ${\rm{k}}\parallel {\rm{c}}$. Polarized light was produced by a wire-grid polarizer with a KRS-5 substrate. The spectral resolution was 0.5 cm^−1^.

For alignment of interstitial hydrogen and deuterium, uniaxial stress was applied parallel to the [100] axis at *T* ≤ 10 K and reference spectra were recorded. After that, with the stress on, the sample was warmed up to a temperature of interest, annealed at this temperature for 10 min and subsequently cooled down to *T* ≤ 10 K. Then new spectra were recorded and compared against the reference one. This procedure was repeated for gradually enhanced temperatures until further annealing did not result in a change of the IR absorption lines discussed below.

### Calculational details

For all density functional theory (DFT) calculations, the PBEsol functional^30^ was employed which, in solids, generally slightly improves the accuracy compared to the traditional PBE^31^. The Vienna *ab initio* simulation package (VASP) code^32–34^, was used with 2 × 2 × 3 supercell comprising 73 atoms (the interstitial ${{\rm{H}}}_{i}^{+}$/${{\rm{D}}}_{i}^{+}$ and 72 host atoms) and an increased cutoff energy of 520 eV. For Ti, *s*-orbital semi-core states were included explicitly. A 4 × 4 × 4 *k*-point grid has been used for all calculations except for LVMs where one special *k*-point at (1/4, 1/4, 1/4) in reciprocal coordinates was used, following the gist of Refs^35,36^. For calculation of vibrational frequencies the linear-response theory based approach, as implemented in the VASP code, was applied. In order to find the transition state (saddle point) for the motion of ${{\rm{H}}}_{i}^{+}$/${{\rm{D}}}_{i}^{+}$ the climbing image nudged elastic band (cNEB) method^37^ was employed.

## Results

**Figure 1:**
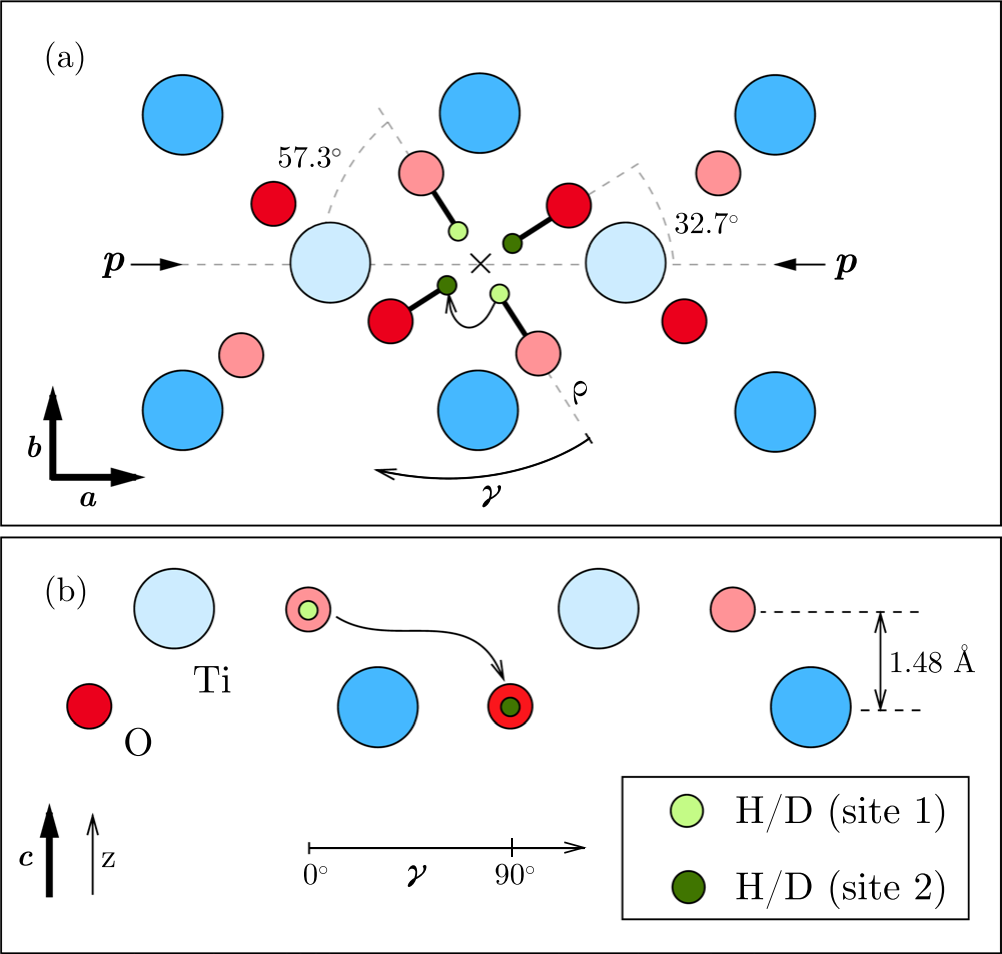
(**a**) The (001) projection of the rutile TiO_2_ lattice with calculated most stable positions of interstitial H/D. The *c*-channel axis is denoted as “×”. The equivalent lattice atoms and hydrogen sites marked by different colors lay in the adjacent planes stacked in the *c*-direction. Shown is also the direction of the applied stress *p*. (**b**) Cylindrical projection around the channel axis along the angle *γ*. Only the lattice sites closest to the channel axis are shown. The elementary diffusion jump is schematically shown. For details on the diffusion path cf. Fig. 6.

Figure 1(a) shows the rutile lattice projected onto the (001) plane with the relative distances between the atoms presented in accordance with our *ab initio* calculations which are described below. The conventional tetragonal elementary cell contains two chemical units. All O–H bonds lie in the figure plane, i.e. “perpendicular” to *c*. The rutile lattice has a *P*42/*mnm* space group which implies that four equivalent orientations of a O–H bond are possible, as indicated in the figure. Two of the O–H bonds (site 1) are rotated by *α* = 32.7° relative to the *a* axis, whereas the other two (site 2) are rotated by the same angle relative to *b*. The pairs of oxygen atoms are stacked along ***c*** with a displacement of *d* = 1.48 forming a preferential diffusion channel for hydrogen. The elementary diffusion step along the channel is a jump between the sites 1 and 2^38^. For a better visualization the radial projection of the lattice structure with respect to the channel axis is shown in Fig. 1(b).

**Figure 2:**
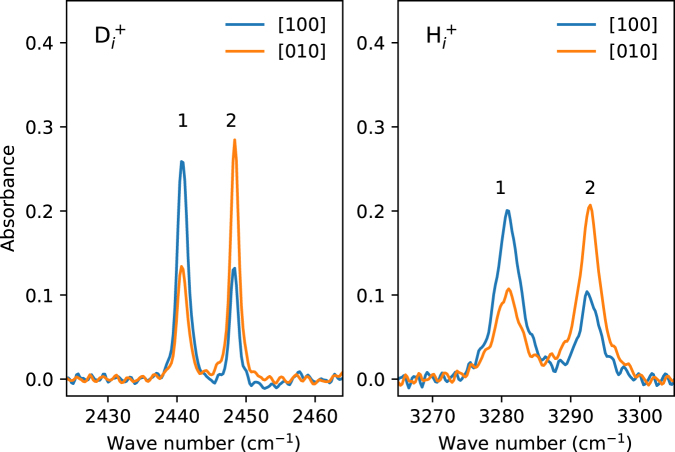
Sections of polarized absorption spectra of the TiO_2_ sample under a uniaxial stress of 227 MPa, ${p}\parallel \mathrm{[100]}$, applied at *T* ≤ 10 K.

Figure 2 shows sections of IR absorption spectra of the TiO_2_ sample subjected to the uniaxial stress of 227 MPa, ${p}\parallel \mathrm{[100]}$, applied after the sample was cooled down to *T* ≤ 10 K. As a result the LVMs of ${{\rm{H}}}_{i}^{+}$ and ${{\rm{D}}}_{i}^{+}$ split into two components each of which is polarized with respect to the direction of the stress. Relative intensities of the split-off components labeled 1 and 2 are close to the values expected from the geometrical model of the defect presented in Fig. 1: ${\cot }^{{\rm{2}}}\alpha$:1 = 2.4:1 and 1:${\cot }^{{\rm{2}}}\alpha$ = 1:2.4, respectively. Deviation from the experimental values of approximately 2:1 and 1:2.2 we explain by uncertainty of the calculated value of the angle and/or the non-idealities in the position of the polarizer.

In linear approximation (Hooke’s law), the frequency of a non-degenerate LVM shifts with the stress *σ*_*ik*_ as

1 $${\rm{\Delta }}\omega =\sum _{i,k}{A}_{ik}{\sigma }_{ik},$$

where *A*_*ik*_ is the second-rank stress tensor. In the assumption that the two of the main axes of the stress tensor ${A}_{\parallel }$ and *A*_⊥_ of ${{\rm{H}}}_{i}^{+}$ $({{\rm{D}}}_{i}^{+})$ are aligned parallel and perpendicular to the O–H bond, we obtain that the frequencies of the LVMs due to the sites 1 and 2 shift with the stress as

2 $$\begin{array}{c}{\rm{\Delta }}{\omega }_{1}=({A}_{\parallel }{\cos }^{2}\alpha +{A}_{\perp }{\sin }^{2}\alpha )\sigma \\ {\rm{\Delta }}{\omega }_{2}=({A}_{\parallel }{\sin }^{2}\alpha +{A}_{\perp }{\cos }^{2}\alpha )\sigma ,\end{array}$$

where *σ* is the value of stress. With the frequencies of the split-off components presented in Fig. 2 we obtain that for ${{\rm{H}}}_{i}^{+}$ the diagonal components of the stress tensor are ${A}_{\parallel }^{{\rm{H}}}=-65$ and ${A}_{\perp }^{{\rm{H}}}=60$ cm^−1^/GPa, whereas for ${{\rm{D}}}_{i}^{+}$ they are reduced by approximately a factor of $\sqrt{{\rm{2}}}$ to ${A}_{\parallel }^{{\rm{D}}}=-42$ and ${A}_{\perp }^{{\rm{D}}}=38$ cm^−1^/GPa. The LVM of ${{\rm{H}}}_{i}^{+}$ in rutile is about an order of magnitude more sensitive to the stress compared to that of hydrogen-related defects in ZnO^39,40^.

The analytical treatment given in^41^ can be used to estimate the energy difference Δ*E* = *E*_2_ − *E*_1_ between the two sites as a function of the LVM splitting under the stress. Employing the expression given in^41^, one gets Δ*E* = ≈ 25 meV for the splitting presented in Fig. 2. As will be shown later, a more accurate value will be derived from both theory and experimental data.

**Figure 3:**
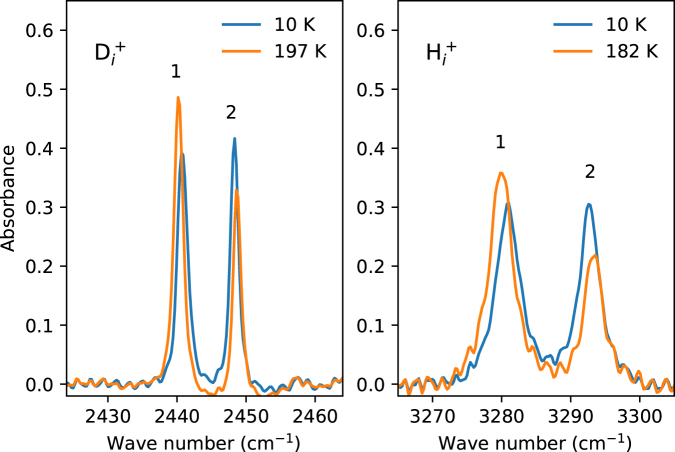
Sections of unpolarized IR absorption spectra measured at *T* ≤ 10 K under a uniaxial stress of 227 MPa, ${p}\parallel \mathrm{[100]}$. The spectra labeled 10 K were measured immediately after applying the stress at *T* ≤ 10 K. Those labeled 197 and 182 K were obtained after annealing under the stress at the corresponding temperatures.

At the temperature of 10 the populations of the sites 1 and 2 remain unchanged since the thermal energy is insufficient to overcome the activation barrier. To investigate the dynamics of the diffusion motion of hydrogen, the sample under the stress was annealed isochronally at elevated temperatures. After annealing at the temperature of interest for 10 min, it was cooled down again to 10 K and polarized spectra were measured. The procedure was then repeated for consecutively higher annealing temperatures. The annealing results in reoccupation of the sites in favor of the one with lower energy as illustrated in Fig. 3, where the absorption spectra before and after annealings at 197 and 182 K are shown.

To extract the ratio of the occupation numbers of the states 1 and 2, the pair of hydrogen (deuterium) peaks (see Fig. 3) was fitted by a sum of two Lorentzian functions of equal full width at half maximum. The occupation ratio was then computed as the quotient of the corresponding Lorentzian amplitudes.

According to the Boltzmann statistics the ratio of equilibrium populations of nonequivalent sites is ${\tilde{n}}_{1}/{\tilde{n}}_{2}=\exp ({\rm{\Delta }}E/kT)$. It is achieved via jumps of ${{\rm{H}}}_{i}^{+}$ $({{\rm{D}}}_{i}^{+})$ between the two sites if sufficient time is provided.

To extract the parameters governing the diffusion of hydrogen along the ***c*** channel, we employ a first order kinetics model. In the framework of this model, the transition rates between two inequivalent hydrogen sites in the stressed sample are given as

3 $${r}_{i}={{\rm{\nu }}}_{0}\exp \,(-{E}_{i}/kT),$$

with *E*_1,2_ = *E*_*a*_ ± Δ*E*/2, where *E*_*a*_ is the activation energy without stress. The time evolution of the occupation numbers is then described by the following system of coupled differential equations:

4 $$\begin{array}{rcl}{\dot{n}}_{1} & = & -\,{r}_{1}{n}_{1}+{r}_{2}{n}_{2}\\ {\dot{n}}_{2} & = & +\,{r}_{1}{n}_{1}-{r}_{2}{n}_{2}\end{array}$$

The solution to these equations is

$$\frac{{n}_{i}(t)-{\tilde{n}}_{i}}{{n}_{i}\mathrm{(0)}-{\tilde{n}}_{i}}={e}^{-\lambda t},\,\quad {\tilde{n}}_{i}=\frac{{r}_{1}{r}_{2}[{n}_{1}\mathrm{(0)}+{n}_{2}\mathrm{(0)]}}{{r}_{i}({r}_{1}+{r}_{2})},$$

where *λ* = *r*_1_ + *r*_2_ and ${\tilde{n}}_{i}$ is the equilibrium occupation (*i* = 1, 2). For the time evolution of the occupation ratio at a given annealing temperature one obtains

5 $$\frac{{n}_{1}(t)}{{n}_{2}(t)}=\frac{{n}_{1}\mathrm{(0)}({r}_{2}+{r}_{1}{e}^{-\lambda t})+{r}_{2}{n}_{2}\mathrm{(0)}(1-{e}^{-\lambda t})}{{n}_{2}\mathrm{(0)}({r}_{1}+{r}_{2}{e}^{-\lambda t})+{r}_{1}{n}_{1}\mathrm{(0)}(1-{e}^{-\lambda t})}.$$

For *t* → ∞ the ratio converges to the equilibrium value ${\tilde{n}}_{1}/{\tilde{n}}_{2}={r}_{2}/{r}_{1}\equiv {e}^{{\rm{\Delta }}E/kT}$.

The model parameters (activation energy *E*_*a*_, attempt frequency *ν*_0_ and stress-induced energy splitting Δ*E*) were determined using the L-BFGS-B method^42^ by a least squares fit of the experimental ratio *n*_1_/*n*_2_ by the expression (5). The initial value of *n*_1_/*n*_2_ was set to 1. The ratio of two occupations appearing as a result of each annealing stage was taken as an initial value for the next temperature. The effects of cooling down to 10 K and subsequent warming up were disregarded.

**Figure 4:**
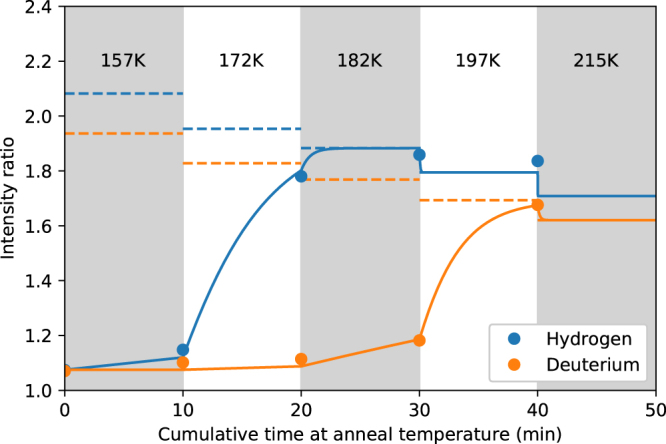
Population ratio *n*_1_/*n*_2_ between the sites 1 and 2 (see Fig. 1) in the course of the annealing procedure under stress of 227 MPa. The circles represent experimental values (see Fig. 2), the solid lines—calculated from Eq. (5) with the best-fit parameters given in Table 1. The dashed lines represent the equilibrium population ratios at the corresponding temperatures.

Figure 4 shows experimental values of *n*_1_/*n*_2_ together with the results of the best fit. It is clearly seen that annealing for 10 min at 172 K (the second step from 10 to 20 min of cumulative annealing time) is not sufficient to reach the equilibrium occupations of the ${{\rm{H}}}_{i}^{+}$ sites. For deuterium the onset temperature of the reorientation shifts up to 197 K (the fourth step from 30 to 40 min of cumulative annealing time). The best-fit parameters of the first order kinetics model given by Eqs (3) and (4) are summarized in Table 1.

**Table 1:** The best fit parameters of the reorientation kinetics of ${{\rm{H}}}_{i}^{+}$ and ${{\rm{D}}}_{i}^{+}$. Values obtained by *ab initio* calculations are given as well. Standard deviations for the fits are stated for ${E}_{a}^{exp}$.

	Hydrogen	Deuterium
${E}_{a}^{exp}$	0.527 ± 0.029 eV	0.584 ± 0.004 eV
${E}_{a}^{DFT}$	0.41 eV	0.44 eV
${\nu }_{0}^{exp}$	3.9 × 10^12^s^−1^	2.37 × 10^12^s^−1^
${\nu }_{0}^{DFT}$	8.4 × 10^12^s^−1^	7.8 × 10^12^s^−1^
Δ*E* ^*exp*^	10 meV	9 meV
Δ*E* ^*DFT*^	8 meV	8 meV
*δE* ^*DFT*^	0.13 eV	0.09 eV

Previous experiments on the diffusion of hydrogen in rutile TiO_2_ were performed at elevated temperatures up to 700 °C^17^. The diffusion constant of hydrogen was found to be strongly anisotropic and equal to ${D}_{\parallel }=1.8\times {10}^{-3}\exp (-0.58\,\mathrm{eV}/kT)$ and *D*_⊥_ = 3.8 × 10^−1^exp(−1.28 eV/*kT*) cm ^2^/s for the directions parallel and perpendicular to the *c* axis, respectively.

**Figure 5:**
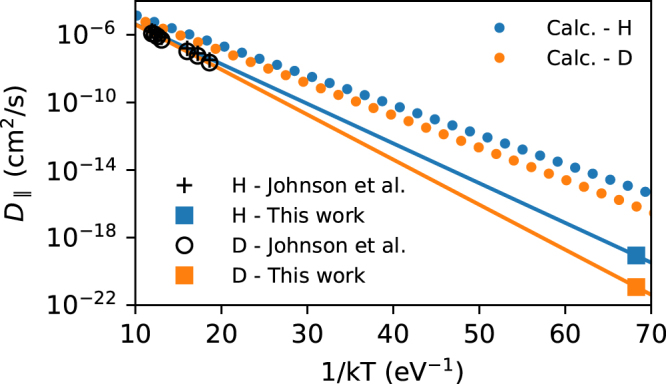
The cumulated diffusion coefficients for hydrogen and deuterium. Dotted lines represent the theoretical values obtained from Eq. (7).

Figure 5 displays the diffusion constants for ${{\rm{H}}}_{i}^{+}$ and ${{\rm{D}}}_{i}^{+}$ taken from Ref^17^. depicted together with the value derived from this study at 170 K. The latter was calculated using the experimentally obtained values of the transition rate *τ* ^−1^ = ν_0_exp(−*E*_*a*_/*kT*) for ${{\rm{H}}}_{i}^{+}$ and and ${{\rm{D}}}_{i}^{+}$, respectively. As the diffusion of hydrogen along the [001] direction occurs by a jump to a neighboring site lying in the adjacent (001) plane separated from the initial one by the distance *d*, the one-dimensional model is applicable and diffusivity is given by^43^.

6 $${D}_{\parallel }=\frac{{d}^{2}}{2\tau }.$$

For 170 K the values of 8.7 × 10^−20^ and 1.210^−21^ cm^−2^/s are derived for ${{\rm{H}}}_{i}^{+}$ and ${{\rm{D}}}_{i}^{+}$ respectively. Based on Fig. 5, the diffusion constants covering both high and low temperature ranges of ${{\rm{H}}}_{i}^{+}$ and ${{\rm{D}}}_{i}^{+}$ in TiO_2_ along the *c* axis are described by

$$\begin{array}{ll}{D}_{\parallel }^{{\rm{H}}} & =\,9.4\times {10}^{-4}\exp (-0.541\,{\rm{eV}}/kT)\,{{\rm{cm}}}^{{\rm{2}}}/{\rm{s}}\\ {D}_{\parallel }^{{\rm{D}}} & =\,1.9\times {10}^{-3}\exp (-0.614\,{\rm{eV}}/kT)\,{{\rm{cm}}}^{2}/{\rm{s}}\end{array}$$

over more then 12 orders of magnitude. Moreover within the experimental accuracy the combined activation energies of 0.541 eV and 0.614 eV also remain in good agreement with the present low-temperature derived values of 0.527 ± 0.029 and 0.584 ± 0.004 eV for the ${{\rm{H}}}_{i}^{+}$ and ${{\rm{D}}}_{i}^{+}$ migration (‘hopping’) barriers.

## Discussion

In the framework of the transition state theory^44,45^, the diffusion process is modeled as a jump from a stationary state (SS), a minimum of potential energy, to another SS which proceeds over a transition state (TS), a saddle point of potential energy. Under harmonic approximation for the vibrational modes and assumption of quantum mechanical partition function, the transition rate is given by

7 $$\begin{array}{c}r=2\frac{kT}{h}\frac{{\prod }_{i}\sinh \frac{h{\nu }_{i}^{SS}}{2kT}}{{\prod }_{i}\sinh \frac{h{\nu }_{i}^{TS}}{2kT}}{e}^{-({E}_{TS}-{E}_{SS})/kT}\end{array}$$

where *E*_*SS*_ and *E*_*TS*_ are the values of potential energy at initial stationary state and transition state, respectively, and ${\nu }_{i}^{SS}$ and ${\nu }_{i}^{TS}$ are the non-imaginary frequencies of the vibrational modes in these states. A single imaginary frequency is characteristic for the transition state and corresponds to the negative curvature of the potential profile along the diffusion pathway.

In the high temperature limit, this expression reduces to the one provided by the classical theory^46^.

8 $$\begin{array}{c}{r}_{{\rm{c}}l}=\frac{{\prod }_{i}{\nu }_{i}^{SS}}{{\prod }_{i}{\nu }_{i}^{TS}}{e}^{-({E}_{TS}-{E}_{SS})/kT},\end{array}$$

whereas at low temperatures it approaches

9 $${r}_{{\rm{qm}}}=\frac{kT}{h}{e}^{-({E}_{TS}-{E}_{SS}-\delta E)/kT},$$

where

10 $$\delta E=\sum _{i}\frac{h{\nu }_{i}^{SS}}{2}-\sum _{i}\frac{h{\nu }_{i}^{TS}}{2}$$

is the zero-point energy correction. In the classical limit isotope substitution does not affect the activation energy, in contrast to that for the low-temperature expression. The opposite holds for the pre-exponential factor.

To gain further insight into diffusion motion of interstitial hydrogen in rutile TiO_2_ first principles calculations were carried out. The parameters of the elementary cell were optimized with respect to the total energy. Besides the equilibrium case, the computation of the structure under stress were carried out. The Young’s modulus and Poisson’s ratio determined in Ref.^47^ were used to model the strain. The computed energy difference between the sites 1 and 2 under the stress along the [100] direction is shown in Fig. 7 together with the experimental values. As seen, the experimental and theoretical values agree reasonably well which further corroborates the validity of our model.

**Figure 6:**
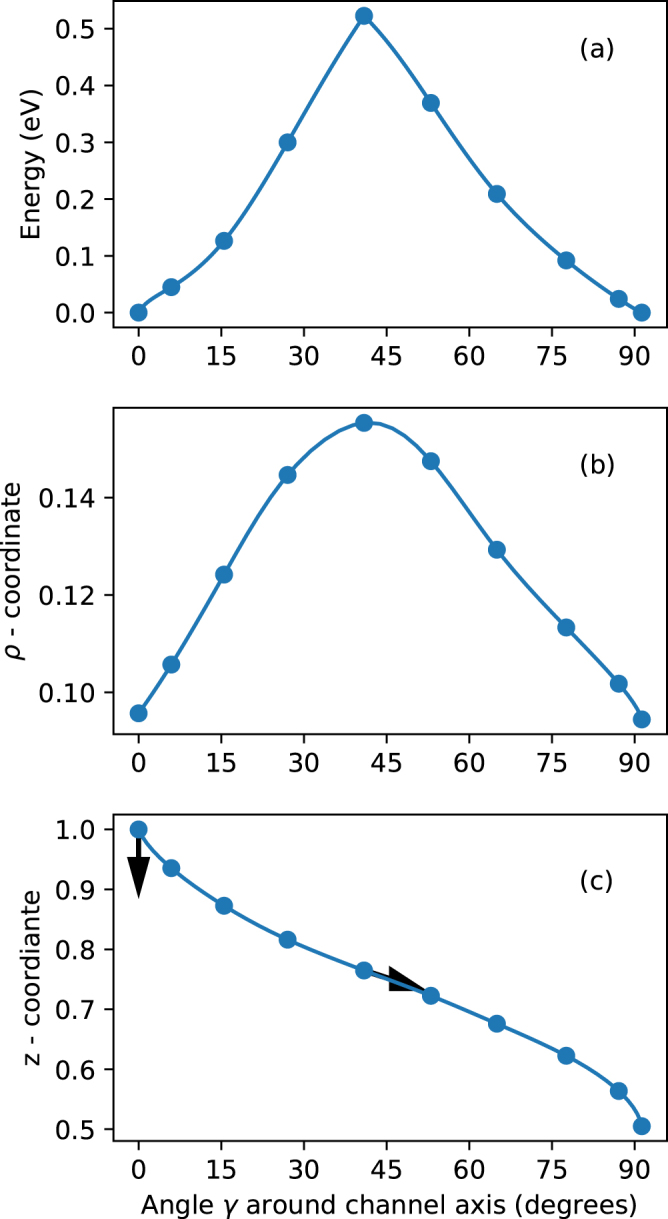
Calculated classical transition path obtained with the cNEB method. The data is shown as a function of the angle *γ* around the channel axis (for definition of cylindrical coordinates *γ*, *ρ*, *z* cf. Fig. 1). The free energy difference to the SS (**a**). The distance from channel axis in units of *a* lattice constant (**b**) and [001] position in units of *c* lattice constant (**c**). The angles of the SS LVM coupling to the migration path (cf. Table 2 *ν*_*w*1_) and the imaginary, TS LVM (cf. Table 2 *ν*_*s*1_) are indicated by arrows.

**Figure 7:**
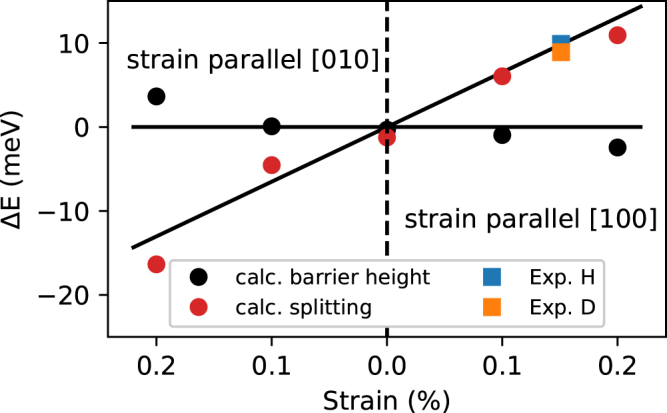
Calculated splitting Δ*E* between the sites 1 and 2 (see Fig. 2) as a function of stress applied along [100] and [010] axis respectively together with the experimental values. The symmetry is a consequence of the crystal structure, deviations reflect numerical noise. Calculated mean barrier defined as ${E}_{TS}-({E}_{S{S}_{1}}+{E}_{S{S}_{2}})/2$.

The transition state (saddle point) for ${{\rm{H}}}_{i}^{+}$ was found by the climbing image nudged elastic band (cNEB) method. The result of the calculation giving a transition barrier height of 0.52 eV is shown in Fig. 6(a). The influence on the change of the calculated mean barrier with strain is shown in Fig. 7. The mean barrier defined as ${E}_{TS}-({E}_{S{S}_{1}}+{E}_{S{S}_{2}}\mathrm{)/2}$ is approximately constant with strain, consistent with the proposed diffusion model. The calculated diffusion pathway is shown in Fig. 6b,c. In addition the vibrational direction of the coupling LVMs on the SS and TS are indicated. In the SS hereby the *ν*_*w*2_ mode (cf. Table 2) and in the TS the *ν*_*s*1_ modes couple to the diffusion pathway. In contradiction to the helical character proposed in^38^, we find that the diffusion paths to both possible hydrogen sites in the adjacent (001) plane are in fact equivalent. Though the path reveals some asymmetry [cf. Fig. 6(a)] this cannot lead to different transition rates for the opposite directions of diffusion.

The linear-response theory, as implemented in the VASP code, was used for calculating the harmonic vibrational frequencies in the ground and transition states. The three energetically highest modes, which correspond to the stretch and wag vibrational mode frequencies of ${{\rm{H}}}_{i}^{+}$ and ${{\rm{D}}}_{i}^{+}$ and mainly contribute to the activation energy^48^ are listed in Table 2. Whereas the calculated frequency of the ground state stretch mode nicely matches the experimental one, the wag modes have escaped an experimental observation so far.

**Table 2:** Calculated (harmonic) local vibrational modes in cm^−1^ for ground and transition states of interstitial hydrogen in titania.

	H (init.)	H (trans.)	D (init.)	D (trans.)
${\nu }_{{s}_{1}}$	3303	977*i*	2403	716*i*
${\nu }_{{w}_{1}}$	1012	1544	729	1104
${\nu }_{{w}_{2}}$	729	1120	552	867

Stretch modes (s) and wag modes (w) are indicated. Imaginary frequencies (*i*) stand for the saddle point. Only the three highest energy modes are shown. Derived values have been calculated using the full vibrational spectrum.

The computed frequencies as well as the energies of the stationary and transition states were substituted into equation (7) and the resulting temperature dependence was fitted in the experimentally used temperature range from 157 to 197 K with the Arrhenius expression (3). This gives activation energies of 0.41 and 0.44 eV and the pre-factors 8.4 × 10^12^s^−1^ and 7.8 × 10^12^s^−1^ for the transition rate of ${{\rm{H}}}_{i}^{+}$ and ${{\rm{D}}}_{i}^{+}$, respectively. The computed activation energy values underestimate the experimental ones. This can be observed also in Fig. 5, where the diffusivity derived from the transition rate using Eq. (6) is shown. The underestimation of the activation energy is common for the PBE/PBEsol-based calculations, and the inclusion of Hartree-Fock exchange may be beneficial. Indeed, test calculations of the saddle point using the HSE06 functional^49,50^ increased the barrier by 0.09 eV which is also consistent with the higher value calculated by Filippone *et al*.^12^ using local exchange (DFT + U).

The different values of the activation energy for ${{\rm{H}}}_{i}^{+}$ and ${{\rm{D}}}_{i}^{+}$ indicate that the reaction kinetics approach quantum character Eq. (9) at the investigated temperatures. In order to verify this we have attempted a two-parametric fit by fixing *ν*_0_ in Eq. (3) to *kT*/*h*. Both the quality of the fit and the values of *E*_*a*_ and Δ*E* remained practically unchanged comparing to the three-parameter fit, which corroborates our conclusion.

In Ref.^23^ the reorientation kinetics for the B-H complex in silicon was studied. In contrast to the present results, the pre-exponential factor for hydrogen was found to be about two orders of magnitude less than that for deuterium, both values being much lower than *kT*/*h*. The authors assumed that quantum tunneling may play an essential role in the considered kinetic process. They applied the Flynn-Stoneham theory^51^ and came to the conclusion that it satisfactory explains the obtained results. As no similar anomalies were revealed in the current study, we suggest that the effect of quantum tunneling is negligible and the diffusion obeys the semiclassical model Eq. (9).

As discussed previously, the data on high-temperature diffusion in rutile TiO_2_ are controversial. Neither a difference in the activation energy for hydrogen and deuterium nor a deviation of the pre-exponential factor ratio from the classical inverse square root dependence on isotope mass were found^17^. Comparing with our results, it implies that a transition from the quantum behavior, Eq. (9), to the classical one, Eq. (8), takes place at intermediate temperatures. This conclusion is, however, challenged by measurements of the tritium diffusion^20,21^. On the other hand, the latter provides controversial values for the activation energy of diffusion. Besides, one cannot exclude that the parameters of hydrogen diffusion may depend on the sample quality. In this context, it should be underlined that the high-temperature diffusion experiments^17^ for hydrogen and deuterium were performed on the same samples, as in the current low-temperature study.

## Conclusions

Hydrogen and deuterium motion in rutile TiO_2_ was probed by means of stress-induced dichroism. The stress applied along the [100] direction partially lifts the orientational degeneracy of two pairs of orthogonal O–H bonds with the rate of 0.04 eV/GPa. Assuming a linear response, this results in a splitting of the corresponding local vibrational modes by 53 and 35 cm^−1^/GPa for the ${{\rm{H}}}_{i}^{+}$ and ${{\rm{D}}}_{i}^{+}$ defects, respectively. It was found that the activation energy of diffusion along ***c***-channel for the two isotopes is 0.53 (hydrogen) and 0.58 eV (deuterium).

The *ab initio* calculations were performed as a part of this study. It is shown that the transition state theory with the vibrational frequencies and activation barrier calculated within the framework of DFT yields diffusion rates of hydrogen isotopes in rutile TiO_2_ in reasonable agreement with experimental data.
